# The variance shared across forms of childhood trauma is strongly associated with liability for psychiatric and substance use disorders

**DOI:** 10.1002/brb3.432

**Published:** 2016-01-21

**Authors:** Sean Kristjansson, Vivia V. McCutcheon, Arpana Agrawal, Michael T. Lynskey, Elizabeth Conroy, Dixie J. Statham, Pamela A. F. Madden, Anjali K. Henders, Alexandre A. Todorov, Kathleen K. Bucholz, Louisa Degenhardt, Nicholas G. Martin, Andrew C. Heath, Elliot C. Nelson

**Affiliations:** ^1^ Alcoholism Research CenterWashington University School of MedicineSt. LouisMissouri63110; ^2^ Institute of Psychiatry, Psychology, and NeuroscienceKing's CollegeLondonUK; ^3^ Centre for Health ResearchUniversity of Western SydneySydneyAustralia; ^4^ QIMR Berghofer Medical Research InstituteBrisbaneAustralia; ^5^ National Drug and Alcohol Research CenterUniversity of New South WalesSydneyAustralia

**Keywords:** Childhood trauma, confirmatory factor analysis, substance use disorders

## Abstract

**Introduction:**

Forms of childhood trauma tend to co‐occur and are associated with increased risk for psychiatric and substance use disorders. Commonly used binary measures of trauma exposure have substantial limitations.

**Methods:**

We performed multigroup confirmatory factor analysis (CFA), separately by sex, using data from the Childhood Trauma (CT) Study's sample of twins and siblings (*N* = 2594) to derive three first‐order factors (childhood physical abuse, childhood sexual abuse, and parental partner abuse) and, as hypothesized, one higher order, childhood trauma factor (CTF) representing a measure of their common variance.

**Results:**

CFA produced a good‐fitting model in the CT Study; we replicated the model in the Comorbidity and Trauma (CAT) Study's sample (*N* = 1981) of opioid‐dependent cases and controls. In both samples, first‐order factors are moderately correlated (indicating they measure largely unique, but related constructs) and their loadings on the CTF suggest it provides a reasonable measure of their common variance. We examined the association of CTF score with risk for psychiatric and substance use disorders in these samples and the OZ‐ALC GWAS sample (*N* = 1538) in which CT Study factor loadings were applied. We found that CTF scores are strongly associated with liability for psychiatric and substance use disorders in all three samples; estimates of risk are extremely consistent across samples.

**Conclusions:**

The CTF is a continuous, robust measure that captures the common variance across forms of childhood trauma and provides a means to estimate shared liability while avoiding multicollinearity.

## Introduction

An extensive literature has consistently and robustly demonstrated that exposure to various forms of childhood trauma (e.g., childhood sexual abuse [CSA], childhood physical abuse [CPA]) is associated with risk for diverse psychiatric outcomes (Mullen et al. 1993; Fergusson et al. 1996a; Fergusson and Lynskey 1997; Kessler et al. 1997; Kendler et al. 2000; Molnar et al. 2001; Nelson et al. 2002, 2006; Green et al. 2010; McLaughlin et al. 2010; Scott et al. 2010). Many investigations have relied on binary measures to indicate the presence of a single form of trauma (e.g., Bevilacqua et al. 2012; Grabe et al. 2012a,b). Studies that assessed multiple trauma categories have generally either combined them into a single binary measure or included variables representing different trauma types in regression‐based analyses (e.g., Huang et al. 2012). To the extent that these variables are highly correlated, the latter option increases the risk of unstable results due to multicollinearity.

These practices have other inherent limitations. First, classical and modern test theory emphasize that observed measures (i.e., items), whether binary, categorical or otherwise, contain measurement error (Crocker and Algina 1986) and are thus imperfect “indicators” of the underlying construct of interest. Indicators that contain large measurement errors are less reliable limiting the statistical power to detect significant effects (Crocker and Algina 1986). Second, binary items are frequently used to represent the presence or absence of trauma without providing information about the severity of the trauma experienced. Third, binary items treat the trauma as a unidimensional construct. However, various forms of trauma exist which, although interrelated, may each lie on its own severity continuum. Fourth, when multiple items are added to form a sum score (which often is then dichotomized), the items are equally weighted in that sum. This is equivalent to assuming that each item measures the construct equally well despite the possibility that certain items might comprise more measurement error than others.

To overcome these limitations, childhood trauma can be conceptualized and operationalized within a latent variable framework. Here, observed items are defined as manifestations of latent variables that cannot be directly measured. The latent variables can be “modeled” using various psychometric and/or statistical measurement methodologies including higher order confirmatory factor analysis (CFA). This method uses many items, each of which is given a different weight (i.e., factor loading) that represents the degree to which latent trauma variables are manifest in the observed item. Those components of observed items that are not related to the latent variables are held in the model residuals (to which sampling, measurement, and modeling error all contribute [Raudenbush and Bryk 2002; ]). Thus, this process removes some item‐level measurement error from the latent variables while creating a higher order factor that is multidimensional and continuously distributed along severity continua. Two studies (Scher et al. 2001; Spinhoven et al. 2014) have performed CFA using data collected with the widely used Childhood Trauma Questionnaire‐Short Form, a 28‐item retrospective self‐report measure from which five factors can be derived, each representing forms of abuse and neglect. One report (Scher et al. 2001) found that the original five correlated first‐order factors (Bernstein et al. 2003) provided somewhat better statistical fit than a model that assumed that the five factors were not intercorrelated, but instead loaded onto a single higher order factor. A later report (Spinhoven et al. 2014) found two alternative models had comparable fit to the original five factor model: (i) five correlated first‐order factors loading onto a single latent general factor; and (ii) allowing individual items to load directly onto both the five first‐order factors and a general factor.

The current report hypothesizes that the common variance derived from three distinct types of childhood trauma (i.e., CSA, CPA, and parental partner abuse [PPA]) provides a robust continuous measure of their shared liability. We use CFA to create three first‐order factors, each a continuous measure of a specific form of childhood trauma, and a higher order childhood trauma factor (CTF) derived from their common variance. The availability of childhood trauma exposure data collected with the Christchurch Trauma Assessment (Fergusson et al. 1989, 1996a,b; Fergusson and Lynskey 1997) from adult participants in three large Australian studies enabled the use of one sample to develop the CTF, the second to replicate it, and the third to demonstrate that similar association results are observed with application of factor loadings from the primary sample. To demonstrate the CTF's predictive validity, we examine its association with psychiatric and substance use disorders in the three samples. In post hoc analyses, we show that CTF scores are significantly correlated with measures of other forms of childhood maltreatment (i.e., emotional abuse and neglect) not included in this measure. As we demonstrate here with the CTF, continuous measures of childhood trauma can provide broad construct coverage and excellent predictive power.

## Materials and Methods

### Samples

Detailed descriptions of the Childhood Trauma (CT) Study (Nelson et al. 2010; Sartor et al. 2012), Comorbidity and Trauma (CAT) Study (Conroy et al. 2009; Shand et al. 2011; Nelson et al. 2013), and OZ‐ALC genome‐wide association study (GWAS) (Heath et al. 2011) methods have been reported; summaries are provided below.

### Primary sample – CT Study

Data from a semistructured psychiatric diagnostic assessment conducted 1996–2000 via telephone (Heath et al. 2001) with a large Australian volunteer twin panel (Cohort II, born between 1964 and 1971) were used to ascertain families. High‐risk families were those in which at least one twin endorsed a screening question on CSA (5 total) or CPA (4 total); in control families, no twin endorsed any of these items. Verbal consent, obtained preinterview, was confirmed by return of a signed consent form allowing use of interview data as per procedures approved by the QIMR Berghofer Medical Research Institute (QIMR) Ethics Committee and the Washington University School of Medicine (WUSM) Human Research Protection Office (HRPO). 3407 respondents from 524 high‐risk and 373 control families completed telephone interviews 2003–2008 and provided postinterview consents. Data from 2594 twin and sibling respondents with childhood trauma assessment available (parents [*N* = 813] were not similarly assessed) are reported including 1532 twins (996 female [65.0%]) and 1062 nontwin siblings (625 female [58.9%]). The mean age at interview was 37.2 years (SD 2.3) for twins and 40.6 years (SD 6.3) for siblings.

### Replication sample – CAT Study

The CAT Study is a case–control examination of genetic and environmental factors contributing to liability for opioid dependence. One thousand four hundred and sixty‐eight cases (577 female [39.3%]) were recruited from opioid substitution therapy (OST) clinics in the greater Sydney region and 513 controls (284 female [55.4%]) from geographic areas in proximity to OST clinics who had minimal or no (0–10 times lifetime) opioid misuse (Conroy et al. 2009; Shand et al. 2011; Nelson et al. 2013). Written informed consent was obtained from all participants as per the institutional review board approvals from the University of New South Wales, WUSM, QIMR, and the ethics committees governing the participating clinics. The mean age at interview was 36.4 years (SD 8.6) for cases and 34.7 years (SD 10.6) for controls.

### Replication sample – OZ‐ALC GWAS

A reassessment of participants in a genome‐wide association study (GWAS) (Heath et al. 2011) of alcohol dependence and heaviness of drinking termed the OZ‐ALC GWAS, focused on childhood and adult environmental stressors. The GWAS sample was ascertained through index cases identified via surveys of two large general population Australian twin cohorts (cohort 1, born 1890–1964 [but mostly 1940–1964], and cohort 2 born 1964–1971), (Heath et al. 1997; Knopik et al. 2006) the spouses/partners of cohort 1 twins, (Grant et al. 2007) and an Australian population‐representative sample that ascertained families containing five or more full siblings. Recruitment of families in which a member reported a history of childhood trauma at prior interview was prioritized. Excluding CT Study participants and those with missing Christchurch Trauma Assessment data, a sample of 747 women and 791 men (mean age 53.0 years [SD 8.2]) were retained for the current analyses. Verbal consent was obtained preinterview as per the protocol approved by the QIMR Ethics Committee and the WUSM HRPO.

### Assessments

The computer‐assisted diagnostic interview in the CT Study included the modified Christchurch Trauma Assessment (Fergusson et al. 1996a, 1989; Fergusson and Lynskey 1997; Fergusson et al. 1996b; available on request to interested investigators) which contains detailed questions assessing CPA (14 items, asked separately about mother, father, and other adult members of the household, covering forms of severe physical punishment occurring before age 18 [including whether each endorsed form occurred occasionally or frequently] and punishment‐related injuries), CSA (17 items asking about noncontact, contact and penetrative sexual abuse occurring before age 18 as well as additional questions to determine the frequency of abuse occurring during various age periods), and PPA (19 total items including separate questions about emotional and physical abuse of partner by each parent during the respondent's childhood or adolescence and additional items querying whether police visited the home, respondent and mother left the home, or if the respondent avoided spending time in the home). To demonstrate the utility of the factors, associated risks for lifetime axis I disorders and suicidal thoughts and behavior, assessed via telephone with a modified version of the Semi‐Structured Assessment for the Genetics of Alcoholism (SSAGA), (Bucholz et al. 1994) were examined. When multiple assessments of an outcome were available on a participant, a positive response at any assessment was coded positive. Participants were also asked to complete and return (by mail) self‐report questionnaires that included the modified Neglect Scale (Straus et al. 1995) with three additional items that assessed emotional abuse (EA) embedded within it.

The CAT Study computer‐assisted face‐to‐face diagnostic assessment also included SSAGA‐based (Bucholz et al. 1994) assessment of lifetime axis I disorders and suicidal thoughts and behavior. Modifications made to the Christchurch Trauma Assessment (Fergusson et al. 1989, 1996a,b; Fergusson and Lynskey 1997) to reduce respondent burden for this study included combining maternal and paternal CPA assessments and combining some CSA screening questions reducing the total from 17 to 11 items (see Table S1). A small number of questions assessing neglect (4) and EA (2) were added to the interview during the first year of the study.

The computer‐assisted interview administered via telephone to the OZ‐ALC GWAS sample included the modified Christchurch Trauma Assessment (Fergusson et al. 1989, 1996a,b; Fergusson and Lynskey 1997) and sections from the SSAGA (Bucholz et al. 1994) that assessed lifetime diagnoses of illicit drug dependence and posttraumatic stress disorder (PTSD) and past year diagnoses of nicotine and alcohol dependence. Since all participants had been assessed previously, other lifetime diagnoses were obtained from previous SSAGA (Bucholz et al. 1994) interview data. A positive response at any assessment was again coded positive. The interview included the modified Neglect Scale (Straus et al. 1995) which had four additional items that assessed emotional abuse (EA) embedded within it.

### Statistical analysis (see Supplementary Methods for details of data preparation)

#### Data preparation

CT Study childhood trauma exposure data were examined to identify items with endorsement frequencies insufficient for inclusion in factor analyses (i.e., resulting in problematic empty cross‐tabulated cells). Items assessing more severe forms of abuse within each domain, and those with nonbinary ordinal responses were determined to have inadequate endorsement for inclusion as initially operationalized. Several steps were taken to address these issues. Low endorsement items assessing similar forms of abuse were combined. Items with nonbinary ordinal responses were recoded to be binary variables representing endorsement at any level. In the interview's CPA section, participants were asked parallel series of questions about abuse by mother, father, and other adult household members. Items assessing parallel sets of items were combined across parents; those covering nonparent household members were dropped due to extremely low endorsement. These changes reduced the number of items included in the first‐order factor analysis to 37 (CPA = 9, CSA = 13, PPA = 15).

#### Multigroup second‐order factor model (CT Study sample)

We hypothesized that the data could be modeled with three first‐order latent factors (CPA, CSA, and PPA) and that the covariance among the first‐order factors can be represented by one second‐order factor, which we term the CTF (see Fig. S1). These analyses were conducted with CFAs appropriate for ordered categorical data (Muthén 1984; Lubke and Muthen 2004) that used mean and variance adjusted weighted least squares estimation (WLSMV). The CFAs, conducted in Mplus 5.2, (Muthen and Muthen 1998) accounted for missing observations. Preliminary CFAs were conducted in female and male groups separately to ensure the second‐order factor model fit the data adequately in both groups. Standard errors were adjusted for nonindependence of observations due to familial clustering. Adequacy of model fit was determined using the comparative fit index (CFI), Tucker‐Lewis index (TLI), and root mean square error of approximation (RMSEA).

To be certain that the same trauma constructs were being measured in women and men, we tested whether the structure of the factors demonstrated sufficient measurement invariance (MI) with respect to sex (Meredith 1993; Lubke and Muthen 2004). Following the framework of Muthen and Muthen (Muthen and Muthen 1998), we fit a baseline model that allowed the factors' measurement structures to differ across the female and male groups. Specifically, the baseline model imposed the following constraints across the female and male groups: (i) thresholds and factor loadings were freely estimated for the first‐order factors; (ii) the loadings for the second‐order factor were also freely estimated; (iii) scale factors were set to one in both groups; (iv); intercepts (means) for the first‐order factors were set to zero in both groups and their variances were set to one; and (v) the mean and variance of the second‐order factor were set to zero and one, respectively. Then, we fit more restrictive (nested) models that equated the measurement structures across male and female groups (factor loadings and thresholds for the first‐order factors; factor loadings and intercepts for the second‐order factor) and tested for significant differences in the fit of the more restrictive models to the fit of the baseline model using the difference test option in Mplus (for which the only meaningful statistic provided is the *P*‐value) (Muthen and Muthen 1998).

#### Replication analyses (CAT Study sample)

Less preparation was required for these data due to the modifications made to Christchurch Trauma Assessment (Fergusson et al. 1989, 1996a,b; Fergusson and Lynskey 1997) for the project (detailed above). Nonbinary ordinal responses were similarly recoded and some low endorsement items were again combined. Changes reduced the number of items included in the factor analysis to 32 (CPA = 9, CSA = 8, PPA = 15). Multigroup CFAs were then similarly performed as was done for the CT Study data. Due to the streamlined nature of the trauma assessment, combining the CT Study and CAT Study samples in a single multigroup model was not feasible.

#### Replication analyses (OZ‐ALC GWAS)

Items were recoded as described for the CT Study sample. Factors were created using the CT Study loadings with results standardized to match the CT Study estimated means (including a higher mean for the CSA factor in women). The loadings from the CT Study were then applied to the standardized first‐order factor scores to calculate second‐order factor scores.

#### Regression analyses

To demonstrate the predictive validity of the CTF, we conducted regression analyses in SAS version 9.4 (SAS Institute, Inc. 2013); analyses of CT Study and OZ‐ALC GWAS sample data used robust variance estimators to adjust 95% confidence intervals for the presence of familial clustering. Logistic regression analyses were used to examine the CTF‐associated risk for outcomes, in models that controlled for gender. For each overall sample, the CTF scores were first standardized in SAS to have means of zero and standard deviations (SD) of one. Odds ratios thus calculate risks associated with a one‐unit (i.e., 1 SD) difference in CTF score. Multinomial logistic regression was similarly used to determine risk, controlling for gender, for the number of licit and illicit drug dependence diagnoses associated with the CTF score. Post hoc analyses were performed in each sample's data that: (i) included neglect and EA in the regression models; and (ii) examined the correlation of the CTF score with neglect and EA (see Supplementary Methods).

## Results

Endorsement frequencies of the items used in the factor analyses are displayed in Table S1. The values in the CT Study and OZ‐ALC GWAS samples are very similar throughout. While overall item endorsement was highest in the CAT Study sample, relative frequencies in the three samples are quite consistent.

### Factor analyses, CT Study

The baseline multigroup second‐order CFA fit the CT Study data well (CFI = 0.976, TLI = 0.974, RMSEA = 0.029). Constraining the first‐order factor loadings and thresholds and the higher order factor loadings and intercepts across gender indicated that measurement invariance (MI) did not hold (*P* < 0.001). However, partial MI was obtained by freeing the loading of a single item assessing attempted or completed anal sex, and freeing the intercept for the first‐order CSA factor (*P* = 0.091). In addition, fit indices suggested the model with partial MI also fit the data well (CFI = 0.981, TLI = 0.981, RMSEA = 0.025). For the first‐order factors, standardized factor loadings (see Table S2) ranged from, 0.69–0.94 for CPA, 0.58–0.97 for CSA and 0.69–0.96 for PPA. Standardized Loadings on the second‐order factor (CTF) were 0.82 for CPA, 0.69 for PPA, and 0.50 for CSA (Table 1).

**Table 1:** Standardized factor loadings of first‐order factors childhood physical abuse, parental partner abuse, and childhood sexual abuse on second‐order childhood trauma factor

First‐order factors	CT Study		CAT Study	
	Factor loading (SE)		Factor loading (SE)	
	M	F	M	F
Childhood physical abuse	0.82 (0.05)	0.82 (0.05)	0.84 (0.04)	0.84 (0.04)
Parental partner abuse	0.69 (0.04)	0.69 (0.04)	0.64 (0.03)	0.64 (0.03)
Childhood sexual abuse	0.50 (0.04)	0.50 (0.04)	0.29 (0.05)	0.58 (0.04)

CT, childhood trauma; CAT, comorbidity and trauma; SE, standard error.

These results suggested the differences in the factor structures of the CFA models by gender were minimal and anticipated. Specifically, the anal sex item had a significantly lower loading on the CSA factor in women (0.69) compared to men (0.97) while the intercept of the CSA factor (i.e., mean level on the CSA factor after controlling for the second‐order factor) was significantly higher in the women (0.50) compared to men (0.00). Given these minimal differences, it is apparent that the interview items assessed comparable abuse constructs in females and males.

### Factor analyses, CAT Study

The baseline multigroup second‐order CFA model also fit the CAT Study data well (CFI = 0.97 TLI = 0.97; RMSEA = 0.06). As above, constraining the first‐order factor loadings and thresholds and the second‐order factor loadings and intercepts across groups resulted in a significant deterioration in model fit (*P* < 0.05). However, partial measurement invariance was again obtained by freeing the loading for the anal sex item and the intercept for the first‐order CSA factor, and additionally by freeing the loading of the first‐order CSA factor. The fit indices again suggested the model with partial MI fit the data well (CFI = 0.955, TLI = 0.972, RMSEA = 0.089). Overall, the CFA results in CAT Study are also consistent with assessment of comparable abuse constructs in males and females. Factor loadings on the first‐ and second‐order factors are shown in Tables S2 and 1 respectively. For the first‐order factors, standardized factor loadings ranged from, 0.72–0.94 for CPA, 0.55–0.94 for CSA and 0.64–0.99 for PPA. Standardized Loadings on the second‐order CTF were 0.84 for CPA, 0.64 for PPA, and 0.58 for CSA in women and 0.29 in men.

The correlations of the first‐order factors are shown in Table 2. CPA and PPA factors had the highest correlations in both men and women. In general, the CSA factor was more highly correlated with the other factors in women than men. In no case were the comparable correlations higher in men than women. Overall, the moderate correlations of the first‐order factors observed in both men and women imply that they are measuring largely unique constructs. The fairly balanced loadings of the first‐order factors on the second‐order factor indicate that the CTF represents a reasonable estimate of their common variance.

**Table 2:** Correlation of the first‐order factors in male (above diagonal) and female (below) participants of each investigation

First‐order factors	CPA	PPA	CSA
CT Study			
Childhood physical abuse	–	0.72*	0.57*
Parental partner abuse	0.72*	–	0.49*
Childhood sexual abuse	0.52*	0.41*	–
CAT Study			
Childhood physical abuse	–	0.63*	0.29*
Parental partner abuse	0.63*	–	0.24*
Childhood sexual abuse	0.55*	0.40*	–
OZ‐ALC GWAS			
Childhood physical abuse	–	0.45*	0.24*
Parental partner abuse	0.46*	–	0.14**
Childhood sexual abuse	0.30*	0.32*	–

*P < 0.001; **P = 0.001.

CT, childhood trauma; CAT, comorbidity and trauma; CPA, childhood physical abuse; PPA, parental partner abuse; CSA, childhood sexual abuse; GWAS, genome‐wide association study.

### Association with psychiatric and substance use disorders

Across all three samples, CTF scores were associated with significant risk for lifetime individual licit and illicit substance dependence diagnoses, conduct disorder, depression, PTSD, and suicide attempt (Table 3). Examinations focusing on the numbers of licit and illicit drug dependence diagnoses found consistent evidence (Table 4) that CTF scores are also associated with incrementally greater magnitude of risk for larger total numbers of diagnoses. The CTF is thus a unitary measure with strong psychometric properties that captures covariance common to three types of adversity and is associated with significant risk of psychiatric and substance use disorders indicative of its strong predictive validity.

**Table 3:** Regression analyses examining risk^a^ associated with CTF score

Outcome	Odds ratio (95% confidence interval)		
	CT Study	CAT Study	OZ‐ALC GWAS
MDD	1.69 (1.55–1.84)	1.55 (1.40–1.70)	1.64 (1.46–1.85)
PTSD	2.79 (2.45–3.17)	2.06 (1.86–2.29)	2.39 (2.06–2.77)
Conduct disorder	2.06 (1.77–2.40)	2.02 (1.83–2.24)	1.68 (1.48–1.92)
Suicide attempt	2.07 (1.78–2.42)	1.68 (1.47–1.92)	1.91 (1.65–2.21)
Substance dependence diagnoses			
Alcohol	1.35 (1.22–1.49)	1.59 (1.45–1.75)	1.32 (1.17–1.48)
Nicotine	1.43 (1.31–1.57)	1.47 (1.33–1.62)	1.40 (1.25–1.58)
Cannabis	1.62 (1.38–1.89)	1.52 (1.38–1.66)	1.59 (1.38–1.83)
Stimulant	1.75 (1.41–2.18)	1.55 (1.41–1.70)	1.68 (1.37–2.07)
Sedative	2.33 (1.67–3.25)	1.49 (1.35–1.65)	1.95 (1.36–2.80)
Opiate	2.02 (1.54–2.64)	1.75 (1.56–1.97)	2.00 (1.52–2.64)
Cocaine	1.74 (1.26–2.39)	1.43 (1.29–1.59)	2.53 (1.69–3.77)

a Odds ratios estimate risk associated with a one SD increment in the CTF score adjusted for sex.

CTF, childhood trauma factor; CT, childhood trauma; CAT, comorbidity and trauma; GWAS, genome‐wide association study; MDD, major depressive disorder; PTSD, posttraumatic stress disorder.

**Table 4:** Multinomial regression examining risk for drug dependence diagnoses associated with CTF score (controlling for sex)

CT Study		CAT Study		OZ‐ALC GWAS	
Total	Odds ratio (95% CI)	Total	Odds ratio (95% CI)	Total	Odds ratio (95% CI)
Licit drug dependence diagnoses					
2	1.69 (1.48–1.93)	2	2.02 (1.78–2.30)	2	1.64 (1.41–1.92)
1	1.33 (1.21–1.47)	1	1.51 (1.34–1.70)	1	1.34 (1.15–1.55)
0	1.00 (reference)	0	1.00 (reference)	0	1.00 (reference)
Illicit drug dependence diagnoses					
≥3	2.42 (1.82–3.21)	≥3	2.51 (2.16–2.92)	≥3	2.47 (1.75–3.48)
2	1.99 (1.46–2.71)	2	1.83 (1.55–2.15)	2	1.82 (1.37–2.42)
1	1.46 (1.24–1.72)	1	1.48 (1.25–1.76)	1	1.49 (1.29–1.72)
0	1.00 (reference)	0	1.00 (reference)	0	1.00 (reference)

CTF, childhood trauma factor; CT, childhood trauma; CAT, comorbidity and trauma; GWAS, genome‐wide association study.

## Discussion

This study developed and tested a continuous measure of the variance shared across three forms of childhood adversity. The factor structure of the CTF is consistent across data from two samples with markedly different ascertainment strategies and characteristics. In addition, applying the factor loadings from the primary (CT Study) sample to a third sample yielded a similar pattern of association with psychiatric and substance use disorder outcomes. As opposed to commonly used categorical measures of childhood trauma, the CTF distills risk common to several types of childhood adversity to create a single continuous measure that is free of some components of measurement error inherent in the individual observed items and provides wider coverage of the childhood trauma construct. These advances reduce the potential for multicollinearity which occurs when multiple binary measures of trauma are used in a single regression analysis while providing greater statistical power than categorical measures (Viel et al. 2005).

The development of the CTF builds on evidence that measures of childhood sexual and physical abuse and other family adversities are not unitary constructs but rather are interdependent, correlated measures of contextual risk. A study (Green et al. 2010) that used data from the National Comorbidity Survey Replication (NCS‐R) to examine associations of multiple indicators of childhood interpersonal loss and trauma with first onset of psychiatric and substance use disorders offers support for this premise. Descriptive analyses found that measures of parental dysfunction, family violence, and physical and sexual abuse and neglect loaded on a single factor. In predictive analyses, however, individual binary variables were used, and these had a subadditive effect on risk, indicating that each additional adversity increased risk, but to a decreasing extent. Importantly, while childhood adversity increased risk for all classes of disorder, little evidence of specificity of effects was observed (Green et al. 2010). Those findings, in combination with other evidence that childhood adversities do not occur in isolation but tend to cluster (Kessler et al. 1997; Dong et al. 2004; McCutcheon et al. 2010), highlight the importance of considering broader measures of childhood adversity rather than individual events.

The current report found that the first‐order CPA and PPA factors had somewhat higher loadings on the CTF than were seen for the CSA factor. The examination (Green et al. 2010) of NCS‐R data which included a broader range of childhood adversities operationalized as binary measures found a similar pattern of loadings for CPA, family violence, and CSA. The indirect hierarchical model of CTQ‐SF data also found the CSA factor had the lowest loading on their general childhood trauma factor (Spinhoven et al. 2014). Interestingly, the highest loadings were for physical neglect, emotional abuse, and emotional neglect, three forms of childhood trauma not included in the CTF. The moderate correlations we observed between our first‐order trauma factors suggest each represents a largely distinct form of adversity. A somewhat lower correlation of the CSA factor with the other forms of trauma in males was particularly apparently in the CAT Study (in which the loading of the CSA factor on the CTF was also lower in males) and the OZ‐ALC GWAS sample. The ascertainment of these samples on the basis of substance dependence diagnoses may be contributing to these findings. Further investigation may be necessary to determine other contributing factors. The CTF demonstrated good measurement invariance as a function of gender and consistent factor structure in the CAT Study replication sample.

The estimates of increased risk for psychiatric and substance use disorders associated with CTF score in our three disparate samples are remarkably consistent. Among nonsubstance‐related psychiatric outcomes, the strongest risk was noted for PTSD; ORs varied from 2.79 (95% CI 2.45–3.17) in the CT Study to 2.06 (95% CI 1.86–2.29) in the CAT Study and 2.39 (95% CI 2.07–2.77) in the OZ‐ALC GWAS. The extremely consistent ORs for substance‐related outcomes across the three samples provides further evidence of the CTF's predictive validity and is particularly noteworthy given the substantial differences in sample ascertainment. Incremental increases in risk were observed in all three samples with the total numbers of licit and illicit substance dependence diagnoses. Although these findings are consistent with prior examinations of the relationships between childhood adversity and psychopathology (Mullen et al. 1993; Fergusson et al. 1996a; Fergusson and Lynskey 1997; Kessler et al. 1997; Kendler et al. 2000; Molnar et al. 2001; Nelson et al. 2002, 2006; Green et al. 2010; McLaughlin et al. 2010; Scott et al. 2010), it is important to remember that our estimates represent risk associated with a one SD increase in CTF score in contrast to most other reports of risk associated with the history of a particular type of trauma exposure. A National Epidemiologic Survey on Alcohol and Related Conditions (NESARC) report (Keyes et al. 2012) that used structural equation modeling to explore the relationship between five childhood trauma factors and latent externalizing and internalizing dimensions of psychopathology argued that individual trauma factors had unique loadings on these dimensions. Their CSA factor had significant positive loadings on both psychopathology dimensions in men and women; their CPA factor loaded only on externalizing disorders in men and internalizing disorders in women. They included parental partner violence and other forms of adversity as covariates in some analyses. One important caveat to their results is that although the NESARC interview examined five forms of childhood trauma using questions taken from the CTQ‐SF (Bernstein et al. 2003) and the Conflict Tactics Scale (Straus 1979) the number of items used for each ranged from two for physical abuse to five for physical neglect.

The replication of the CTF's factor structure from the CT Study, a population‐based twin and family sample screened for trauma exposure, in the CAT Study, a case–control study of opioid dependence, is a major strength of the current report. This replication, coupled with the demonstration that applying factor loadings from the CT Study to OZ‐ALC GWAS data yield extremely similar estimates of association, support the likely utility of the CTF in a variety of samples. However, whether this factorial architecture and distribution of scores can be recovered in general population samples with putatively lower exposure to trauma should be explored. Furthermore, similar results were obtained based on telephone and face‐to‐face interviews. The detailed trauma assessments conducted in the three samples contribute to the robustness of the CTF.

It is important to note that although the CTF is created from items assessing three important types of childhood trauma, it does not include other types of trauma, particularly neglect (i.e., physical, emotional, and supervisory) (Straus et al. 1995) and emotional abuse, which are also important. The substantial differences in the assessments of neglect and EA across our three samples (in particular, the small number of items in the CAT Study) precluded their inclusion as components of the CTF. Nonetheless, we performed a post hoc examination of their correlation with CTF scores (see Table 5) which found consistent, highly significant (*P* < 0.0001) moderate correlations ranging from 0.37 to 0.55. Post hoc regression analyses that included sample‐specific measures of neglect and EA as covariates found that, in all cases, CTF‐associated risks remained significant (see Table S3), generally with only modest reductions in magnitude. Thus, despite the lack of inclusion of either neglect or EA in the CTF, this measure is significantly correlated with both of these constructs and its associated liability is largely retained with their inclusion in regression models. Future investigations should explore their inclusion in a revised measure.

**Table 5:** Correlation of study‐specific measures of emotional abuse and neglect with the CTF score^a^

	CT Study	CAT Study	OZ‐ALC GWAS
Neglect	0.37	0.54	0.49
Emotional abuse	0.39	0.55	0.55

a All values significant (*P* < 0.0001).

CTF, childhood trauma factor; CT, childhood trauma; CAT, comorbidity and trauma; GWAS, genome‐wide association study.

Among the limitations of the current report is its use of retrospective recall of trauma in adult populations. However, the bias in retrospective reports of childhood adversity tends toward false negatives rather than false positives (Hardt and Rutter 2004). Because all samples are Australian and were either ascertained based on self‐report of childhood trauma exposure, or for genetic studies of substance dependence (populations with high prevalence of childhood trauma compared to general population samples), these results are not necessarily generalizable to population‐based samples. We are encouraged by our finding (Agrawal et al. 2012) of a significant interaction in CAT Study data involving the CPA factor and an endocannabinoid receptor (*CNR1*) polymorphism (rs1049353) associated with anhedonia and anhedonic depression that replicated a similar interaction involving a binary measure of CPA and rs1049353 genotype in a general population Missouri twin sample. However, additional research will be necessary to demonstrate generalizability. It is likely that not controlling for case status in regression analyses examining association of the CTF with outcomes impacted estimates of risk. We did so to allow greater comparability across studies and to enable inclusion of opioid dependence as a dependent variable. In addition, our logistic regression results do not definitively address the temporal ordering of observed associations. In some individuals, onsets of some forms of psychopathology (e.g., conduct disorder) may precede that of childhood trauma exposure. We opted to perform regression analyses rather than survival analyses because the CTF provides an estimate of forms of trauma exposure prior to age 18 as opposed to a single discrete event with a well‐defined onset. The Christchurch Trauma Assessment was administered by trained interviewers in a research setting; no attempt has been made to demonstrate its utility in other settings.

In summary, this study used factor analysis to maximize the information available from a detailed assessment of correlated childhood traumatic events to develop a dimensional, robust and broad measure of childhood adversity. Similar measures may be constructed from data in any investigation in which the assessment of each form of childhood trauma is adequate for inclusion in factor analyses. Measures such as the CTF provide increased power and reduce problems of multicollinearity. Future work will test measurement invariance of the CTF across samples in other countries and those more representative of the general population (i.e., with lower prevalence of childhood trauma).

## Conflicts of Interest

None of the authors have a financial or personal conflict of interest.

## Supporting information


**Data S1.** Supplementary Methods.
**Figure S1.** Factor model showing the three first‐order factors (CSA, CPA, and PPA) and one second‐order factor (CTF) developed in CT Study data.
**Table S1.** Prevalence (%) of factor component items by gender in the three samples.
**Table S2.** Factor loadings (SE) by gender in the CT Study and CAT Study samples.
**Table S3.** Regression analyses examining risk* associated with CTF score including control for emotional abuse and neglect.
