# Supplementary material for: The variance shared across forms of childhood trauma is strongly associated with liability for psychiatric and substance use disorders
Source: Brain Behav. 2016 Jan 21;6(2):e00432. doi: 10.1002/brb3.432 (PMC4720689; doi:10.1002/brb3.432)
Supplement: Supplementary file 1 — Data S1. Supplementary Methods. Figure S1. Factor model showing the three first‐order factors (CSA, CPA, and PPA) and one second‐order factor (CTF) developed in CT Study data. Table S1. Prevalence (%) of factor component items by gender in the three samples. Table S2. Factor loadings (SE) by gender in the CT Study and CAT Study samples. Table S3. Regression analyses examining risk* associated with CTF score including control for emotional abuse and neglect. [file BRB3-6-e00432-s001.docx]

**Supplementary Methods**

**Details of data preparation** CT Study: A number of items had endorsement frequencies that were insufficient for inclusion in factor analyses (i.e., that resulted in problematic empty cross-tabulated cells). Various steps were taken to address these issues. In the CPA section, responses to items assessing forms of CPA recorded initially as three level ordinal variables (i.e., none, occasionally, and frequently) were recoded as binary variables that reflected the absence or presence of this form of abuse. Participants were asked parallel series of questions about CPA by mother, father, and other adult household members. Items assessing parallel sets of abuse were combined across parents to indicate the occurrence or absence of that form of abuse by either parent; those covering non-parent household members were dropped due to extremely low endorsement. Items assessing more severe forms of CPA (i.e., being locked in a cupboard; being burned) were combined (see **Supplementary Table 1**) into a single binary variable indicating the presence or absence of either form of abuse. Items assessing severe consequences of CPA that were similarly combined into a single binary variable include: broken bones, going to a doctor for treatment, going to a hospital for treatment, needing treatment, but not receiving it, and missing school as a result (shown as “other injury” in **Supplementary Tables 1 and 2**). CSA items assessing attempted and completed sexual behaviors were combined into a single binary variable (see **Supplementary Table 1**). Numerical items assessing the number of times any CSA occurred during specified time periods were similarly recoded as binary variables to reflect the presence or absence of CSA during those periods. PPA items with responses recorded initially as three level ordinal variables (i.e., none, occasionally, and frequently) were similarly recoded as binary variables that reflected the absence or presence of this form of abuse. Items assessing more severe forms of PPA involving each that were combined into a single binary variable (see **Supplementary Table 1**) including kicking, choking or strangling, and threatening with a weapon.

CAT Study: Similar steps were taken with the CAT Study interview data. Since the interview’s CPA questions assessed abuse by either parent, these data did not need to be merged.

OZ-ALC: Data preparation was identical to that used for the CT Study.

**Neglect and emotional abuse (EA) measures** CT Study: The self-report questionnaire included 28 items assessing neglect that were asked separately about each parent. These items used a 4 point scale to represent responses ranges from strongly agree to strongly disagree. Discrete aspects of neglect that were assessed and the number of questions (in parentheses) for each were as follows: physical (4), emotional (6), cognitive (5), and supervisory (12). Items were initially recoded such that the direction of endorsement was consistent for each. A total score for each aspect of neglect was calculated by summing scores across both parents and then multiplying by one-half. The score for each aspect of neglect was then divided by the number of component questions contributing to it and added to that of the other aspects to yield a total neglect score. The EA score was similarly calculated: the responses to the three EA questions (each had a similar 4-level response) from both parents were added together and then the total was multiplied by one-half. A total of 347 respondents who failed to return completed questionnaires and thus had missing data for these constructs as did additional participants who did not complete some EA (N=17) or neglect items (N=269).

CAT Study: A small number of questions assessing neglect (4) and EA (2) were added to the interview during the first year of the study after the start of data collection. Each of these items was asked as a three level response (i.e., none, occasionally, and frequently). The two EA items referred to abuse within the family, but not specifically by parents. The neglect items inquired about parents or other primary caretakers. For each construct, the total score was calculated as the sum of all item responses. A total of 227 participants did not have data on either construct and an additional 7 respondents were only missing data on neglect.

OZ-ALC: Neglect and EA items largely overlapping with those from the CT Study were included in the interview. Response again used the above 4 level scale and were recorded separately for each parent. The 17 neglect items again assessed physical (3), emotional (4), cognitive (5), and supervisory (5) aspects. A total neglect score was calculated as described above for the CT Study. The EA total score was also calculated as above. A total of 24 participants had missing data on neglect items.

Figure Legend

Supplementary Figure 1 Factor model showing the three first-order factors (CSA, CPA, and PPA) and one second-order factor (CTF) developed in CT Study data


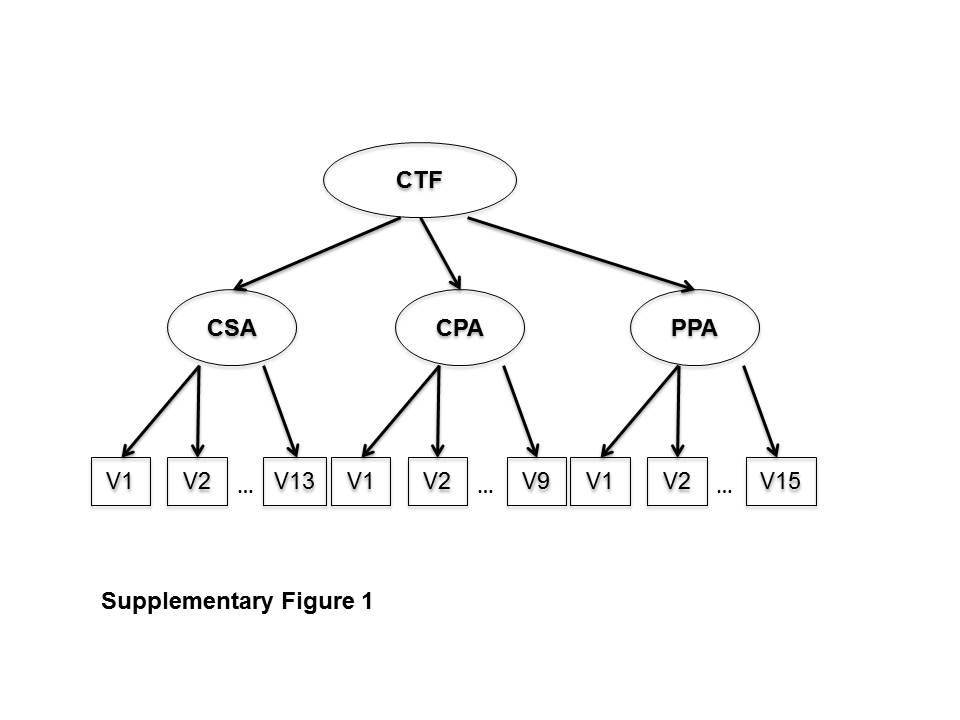


| **Supplementary Table 1** Prevalence (%) of factor component items by gender in the three samples | | | | | | |
| --- | --- | --- | --- | --- | --- | --- |
| Component items by factor | CT Study | | OZ-ALC GWAS | | CAT Study | |
|  | M | F | M | F | M | F |
| **Childhood Physical Abuse** *Father or mother did to respondent*: | | | | | | |
| Hit with fists | 9.9 | 7.6 | 9.2 | 5.9 | 28.3 | 29.4 |
| Hit head or body with stick/object | 9.5 | 8.3 | 15.6 | 12.7 | 23.0 | 24.0 |
| Gave severe beating | 8.2 | 6.9 | 8.6 | 5.5 | 27.0 | 24.5 |
| Kicked | 5.8 | 3.1 | 3.7 | 2.4 | 16.2 | 18.0 |
| Choked/throttled | 3.0 | 1.9 | 2.0 | 1.2 | 11.2 | 14.3 |
| Locked in cupboard/burned^#^ | 2.8 | 1.6 | 2.2 | 3.2 | 9.5 | 8.3 |
| *Parent(s) actions caused:* |  |  |  |  |  |  |
| Bruised/black eye | 15.1 | 13.7 | 14.0 | 12.5 | 32.5 | 31.0 |
| Cut/laceration | 5.2 | 4.7 | 3.9 | 3.5 | 17.9 | 17.3 |
| Other injury^#^ | 3.1 | 2.8 | 2.2 | 3.0 | 16.6 | 18.6 |
| **Childhood Sexual Abuse** | | | | | |  |
| Perpetrator exposed genitals*^1^ | 11.1 | 26.1 | 12.4 | 26.3 | 25.2*^1^ | 49.3*^1^ |
| Perpetrator observed masturbating*^1^ | 6.0 | 10.9 | 8.3 | 10.7 |  |  |
| Touched privates outside clothes*^2^ | 9.8 | 22.3 | 11.6 | 21.2 | 24.3*^2^ | 44.0*^2^ |
| Touched privates inside clothes*^2^ | 8.9 | 20.8 | 9.9 | 18.6 |  |  |
| Forced to touch perpetrators’ genitals*^2^ | 10.2 | 19.6 | 10.9 | 17.6 |  |  |
| Rubbed by privates of perpetrator*^2^ | 7.0 | 17.5 | 8.3 | 17.8 |  |  |
| Had clothes removed^ | 7.3 | 17.6 | 7.0 | 14.9 | ^ | ^ |
| Threaten to force or involve in unwanted sexual activity | 5.2 | 12.5 | 6.4 | 12.2 | 16.5 | 36.7 |
| Attempted/completed intercourse^~^ | 4.9 | 12.4 | 5.1 | 11.9 | 14.8 | 42.5 |
| Attempted/completed oral sex^~^ | 5.4 | 8.4 | 4.2 | 6.8 | 13.3 | 25.3 |
| Attempted/completed anal sex^~^ | 3.4 | 1.8 | 3.7 | 1.3 | 12.3 | 11.2 |
| Any incident occurred ages 6-13 | 12.2 | 26.3 | 12.9 | 25.7 | 23.8 | 43.5 |
| Any incident occurred ages 14-17 | 8.3 | 21.9 | 11.2 | 24.9 | 14.4 | 37.9 |
| **Parental Partner Abuse** *Father’s treatment of mother* | | | | | | |
| Threaten hit or throw something at her | 17.7 | 19.5 | 17.2 | 19.0 | 43.8 | 44.6 |
| Push, grab, or shove her | 16.0 | 18.5 | 15.2 | 17.0 | 41.4 | 43.1 |
| Slap, hit, or punch her | 10.6 | 12.3 | 9.4 | 9.4 | 32.9 | 36.2 |
| Throw, hit, kick or smash something in her presence | 14.5 | 16.0 | 11.6 | 14.9 | 38.0 | 40.4 |
| Kick her/choke or strangle her/threaten her with weapon^#^ | 4.2 | 6.1 | 2.4 | 4.7 | 18.3 | 22.9 |
| Call her names or criticize her | 33.8 | 35.2 | 30.6 | 32.6 | 59.7 | 61.3 |
| *Mother’s treatment of father* | | | | | | |
| Threaten hit or throw something at him | 17.0 | 15.0 | 15.2 | 14.3 | 29.1 | 30.1 |
| Push, grab, or shove him | 9.2 | 7.8 | 8.5 | 7.5 | 22.1 | 24.4 |
| Slap, hit, or punch him | 8.5 | 6.8 | 7.5 | 6.3 | 21.2 | 19.7 |
| Throw, hit, kick or smash something in his presence | 13.6 | 13.8 | 10.8 | 11.5 | 27.8 | 27.9 |
| Kick him/choke or strangle him/ threaten him with weapon^#^ | 3.7 | 2.3 | 2.3 | 2.6 | 10.3 | 10.7 |
| Call him names or criticize him | 34.0 | 31.7 | 27.8 | 31.2 | 49.3 | 53.8 |
| *Consequences* | | | | | | |
| Police came to home | 4.0 | 4.7 | 2.5 | 2.8 | 19.9 | 21.5 |
| Respondent or mother left home because of violence | 6.6 | 7.0 | 3.2 | 4.8 | 20.7 | 24.8 |
| Avoided spending time at home due to problems there | 12.7 | 17.9 | 12.6 | 16.5 | 52.6 | 60.1 |
| *CATS assessment combined items; ^CATS assessment did not include item;  #Separate low endorsement items combined; ^~^Responses to separate questions about attempted and completed forms of abuse combined | | | | | | |

| **Supplementary Table 2** Factor loadings (S.E.) by gender in the CT Study and CAT Study samples | | | | |
| --- | --- | --- | --- | --- |
| Component items by factor | CT Study | | CAT Study | |
| **Childhood Physical Abuse** *Father or mother did to respondent:* | M | F | M | F |
| Hit with fists | .87 (.03) | .87 (.03) | .88 (.02) | .87 (.02) |
| Hit with stick/object | .71 (.05) | .69 (.04) | .84 (.02) | .82 (.03) |
| Gave severe beating | .94 (.03) | .89 (.03) | .91 (.01) | .93 (.02) |
| Kicked | .77 (.05) | .74 (.04) | .87 (.02) | .86 (.02) |
| Choked/throttled | .90 (.04) | .83 (.04) | .89 (.02) | .85 (.03) |
| Locked in cupboard/burned | .78 (.07) | .77 (.05) | .72 (.03) | .79 (.03) |
| *Parent(s) actions caused:* |  |  |  |  |
| Bruised/black eye | .88 (.03) | .87 (.03) | .91 (.02) | .93 (.02) |
| Cut/laceration | .92 (.03) | .89 (.03) | .90 (.02) | .92 (.02) |
| Other injury^#^ | .92 (.04) | .93 (.03) | .94 (.01) | .93 (.02) |
| **Childhood Sexual Abuse** | | | | |
| Perpetrator exposed genitals*^1^ | .94 (.02) | .87 (.02) | .93 (.02)*^1^ | .84 (.02)*^1^ |
| Perpetrator observed masturbating*^1^ | .90 (.02) | .77 (.03) |  |  |
| Touched privates outside clothes*^2^ | .93 (.02) | .92 (.01) | .92 (.01)*^2^ | .91 (.02)*^2^ |
| Touched privates inside clothes*^2^ | .96 (.01) | .94 (.01) |  |  |
| Forced to touch perpetrators’ genitals*^2^ | .97 (.01) | .93 (.01) |  |  |
| Rubbed by privates of perpetrator*^2^ | .96 (.01) | .92 (.01) |  |  |
| Had clothes removed^ | .97 (.01) | .95 (.01) | ^ | ^ |
| Threaten to force or involve in unwanted sexual activity | .95 (.02) | .91 (.01) | .94 (.01) | .89 (.02) |
| Attempted/completed intercourse^~^ | .97 (.01) | .91 (.01) | .93 (.01) | .92 (.02) |
| Attempted/completed oral sex^~^ | .93 (.02) | .83 (.02) | .91 (.01) | .89 (.02) |
| Attempted/completed anal sex^~^ | .97 (.01) | .69 (.05) | .94 (.01) | .82 (.04) |
| Any incident occurred ages 6-13 | .88 (.02) | .77 (.02) | .88 (.02) | .80 (.03) |
| Any incident occurred ages 14-17 | .69 (.04) | .58 (.03) | .65 (.03) | .55 (.04) |
| **Parental Partner Abuse** *Father’s treatment of mother* | | | | |
| Threaten hit or throw something at her | .93 (.01) | .96 (.01) | .94 (.01) | .97 (.01) |
| Push, grab, or shove her | .95 (.01) | .95 (.01) | .97 (.01) | .99 (.01) |
| Slap, hit, or punch her | .95 (.02) | .93 (.01) | .96 (.01) | .96 (.01) |
| Throw, hit, kick or smash something in her presence | .82 (.03) | .84 (.02) | .86 (.02) | .86 (.02) |
| Kick her/choke or strangle her/threaten her with weapon^#^ | .89 (.03) | .85 (.02) | .91 (.01) | .91 (.01) |
| Call her names or criticize her | .78 (.03) | .81 (.02) | .81 (.02) | .78 (.02) |
| *Mother’s treatment of father* | | | | |
| Threaten hit or throw something at him | .87 (.03) | .85 (.02) | .84 (.02) | .87 (.02) |
| Push, grab, or shove him | .84 (.03) | .87 (.02) | .90 (.01) | .86 (.02) |
| Slap, hit, or punch him | .88 (.02) | .84 (.02) | .92 (.01) | .91 (.01) |
| Throw, hit, kick or smash something in his presence | .74 (.04) | .83 (.02) | .81 (.02) | .86 (.02) |
| Kick him/choke or strangle him/ threaten him with weapon^#^ | .82 (.04) | .76 (.04) | .80 (.02) | .80 (.03) |
| Call him names or criticize him | .73 (.03) | .69 (.03) | .70 (.02) | .68 (.03) |
| *Consequences* | | | | |
| Police came to home | .84 (.03) | .76 (.04) | .79 (.02) | .80 (.02) |
| Respondent or mother left home because of violence | .86 (.03) | .83 (.02) | .78 (.02) | .81 (.02) |
| Avoided spending time at home due to problems there | .80 (.04) | .72 (.03) | .64 (.03) | .64 (.03) |
| *CATS assessment combined items; ^CATS assessment did not include item;  ^#^Separate low endorsement items combined; ^~^Responses to separate questions about attempted and completed forms of abuse combined | | | | |

| **Supplementary Table 3.** Regression analyses examining risk* associated with CTF score including control for emotional abuse and neglect | | | |
| --- | --- | --- | --- |
|  | **Odds ratio (95% confidence interval)** | | |
| **Outcome** | **CT Study** | **CAT Study** | **OZ-ALC GWAS** |
| MDD | 1.67 (1.49 – 1.87) | 1.34 (1.18 – 1.52) | 1.49 (1.30 – 1.71) |
| PTSD | 2.48 (2.12 – 2.90) | 1.62 (1.42 – 1.84) | 2.06 (1.71 – 2.47) |
| Conduct disorder | 2.05 (1.69 – 2.49) | 1.69 (1.49 – 1.92) | 1.43 (1.20 – 1.71) |
| Suicide attempt | 1.85 (1.53 – 2.25) | 1.45 (1.21 – 1.73) | 1.53 (1.24 – 1.88) |
| **Substance dependence diagnoses** | | | |
| Alcohol | 1.34 (1.18 – 1.52) | 1.38 (1.22 – 1.56) | 1.28 (1.11 – 1.48) |
| Nicotine | 1.44 (1.29 – 1.61) | 1.27 (1.12 – 1.44) | 1.36 (1.18 – 1.56) |
| Cannabis | 1.66 (1.35 – 2.06) | 1.32 (1.17 – 1.49) | 1.61 (1.32 – 1.98) |
| Stimulant | 1.99 (1.41 – 2.80) | 1.42 (1.26 – 1.60) | 1.69 (1.26 – 2.28) |
| Sedative | 3.12 (1.89 – 5.14) | 1.29 (1.13 – 1.47) | 1.62 (1.06 – 2.46) |
| Opiate | 2.28 (1.58 – 3.28) | 1.50 (1.29 – 1.75) | 1.64 (1.19 – 2.28) |
| Cocaine | 1.82 (1.15 – 2.89) | 1.31 (1.14 – 1.50) | 2.19 (1.25 – 3.84) |
| * Odds ratios estimate risk associated with a one SD increment in the CTF score adjusted for sex and study-specific measures of emotional abuse and neglect | | | |
